# Reply to Okura et al. Comment on “Pu et al. Predicting Postoperative Lung Cancer Recurrence and Survival Using Cox Proportional Hazards Regression and Machine Learning. *Cancers* 2025, *17*, 33”

**DOI:** 10.3390/cancers17050727

**Published:** 2025-02-21

**Authors:** Lucy Pu, Rajeev Dhupar, Xin Meng

**Affiliations:** 1 Department of Bioengineering, University of Pennsylvania, Philadelphia, PA 19104, USA; lpu@seas.upenn.edu; 2 Department of Cardiothoracic Surgery, Wake Forest University, Winston-Salem, NC 27109, USA; rdhupar@wakehealth.edu; 3 Department of Radiology, School of Medicine, University of Pittsburgh, Pittsburgh, PA 15213, USA

Thank you for your thoughtful and constructive comments on our study [1], and we appreciate your insights from a clinical perspective [2].

First, the comments on selecting variables based on univariate significance as mechanistic raise valid concerns. Our approach was motivated by the goal of identifying the most strongly associated predictors for postoperative recurrence and survival. We addressed collinearity issues by incorporating the Variance Inflation Factor (VIF) to avoid overfitting in the multivariate analysis. We acknowledge that certain well-known risk factors may not be fully captured through univariate analysis alone. We agree that incorporating these existing risk factors into the multivariate analysis would enhance the clinical utility of the predictive models. In future analyses, we plan to refine our approach by also including risk factors identified through expert-driven selection.

Additionally, we understand your concern regarding the relatively small test set of 60 patients in our machine learning analysis. To mitigate the impact of the limited sample size, we use cross-validation, which helps ensure the model’s generalizability by evaluating its performance across multiple subsets of the data. While available data constrained our study, the current findings, though preliminary, provide valuable insights. We definitely agree that larger datasets would enhance the predictive accuracy and reliability of machine learning-based models, and we plan to address this limitation in future work by expanding the sample size.

Finally, we appreciate your recommendation to use parametric methods, such as logistic regression or the exponential function, to predict recurrence. We acknowledge that parametric models are less prone to overfitting and offer the potential for extrapolation beyond the time frame of the data. However, at the same time, we would like to note that our choice of using nonparametric methods like Cox regression and machine learning can be beneficial in situations where the underlying distribution of the data is unknown or difficult to model. Nonparametric methods do not rely on strong assumptions about the functional form of the data, making them more flexible and robots in capturing complex, nonlinear relationships that might be missed by parametric models. This can be especially important in clinical applications where patient data can be highly variable. Thus, we believe that combining parametric and nonparametric methods could enhance the overall reliability of predicting recurrence, and we plan to explore the inclusion of parametric approaches in the future.

We are grateful for your feedback and remain committed to refining our methodology. Moving forward, we will consider both parametric and nonparametric methods, as well as expert-driven risk factor selection rather than relying solely on univariate analysis, to enhance the clinical utility and accuracy of our predictions for postoperative lung cancer recurrence. Thank you for your valuable suggestions.
